# Material gain engineering in GeSn/Ge quantum wells integrated with an Si platform

**DOI:** 10.1038/srep34082

**Published:** 2016-09-30

**Authors:** H. S. Mączko, R. Kudrawiec, M. Gladysiewicz

**Affiliations:** 1 Faculty of Fundamental Problems of Technology, Wroclaw University of Technology, Wybrzeże Wyspiańskiego 27, 50-370 Wrocław, Poland

## Abstract

It is shown that compressively strained Ge_1−x_Sn_x_/Ge quantum wells (QWs) grown on a Ge substrate with 0.1 ≤ x ≤ 0.2 and width of 8 nm ≤ d ≤ 14 nm are a very promising gain medium for lasers integrated with an Si platform. Such QWs are type-I QWs with a direct bandgap and positive transverse electric mode of material gain, i.e. the modal gain. The electronic band structure near the center of Brillouin zone has been calculated for various Ge_1−x_Sn_x_/Ge QWs with use of the 8-band ***kp*** Hamiltonian. To calculate the material gain for these QWs, occupation of the L valley in Ge barriers has been taken into account. It is clearly shown that this occupation has a lot of influence on the material gain in the QWs with low Sn concentrations (Sn < 15%) and is less important for QWs with larger Sn concentration (Sn > 15%). However, for QWs with Sn > 20% the critical thickness of a GeSn layer deposited on a Ge substrate starts to play an important role. Reduction in the QW width shifts up the ground electron subband in the QW and increases occupation of the L valley in the barriers instead of the Γ valley in the QW region.

Group IV semiconductors (Si and Ge) are widely applied in electronic devices but their application in light emitters still is very limited because of their indirect bandgap nature. For such semiconductors the electron-hole pair recombination requires phonons in order to preserve momentum. It is the second order recombination process, where the radiative recombination probability is significantly lower than that in the direct recombination processes, which are typical in direct bandgap semiconductors such as III−V alloys. Since the nature of bandgap in group IV semiconductors is the main obstacle to the fabrication of light emitters, the bandgap engineering via strains and/or alloying with other group IV materials is required to make these materials useful for optoelectronic applications. For Si the energy difference between the direct band-gap and the indirect band-gap is very large (2.54 eV), while germanium has a small this energy difference (0.14 eV), hence Ge is a promising material for tuning the nature of bandgap from indirect to direct by strain engineering and/or alloying with other elements of group IV.

In recent years a significant progress has been made concerning the efficiency of luminescence from strain-engineered Ge by inducing high biaxial or uniaxial tensile strain^1^,^2^,^3^,^4^,^5^,^6^,^7^,^8^,^9^. The energy difference between the Γ- and L-valley minima reduces due to the tensile distortion of the Ge lattice, and even if the bandgap is still indirect, larger electron population in the Γ-valley becomes available for efficient direct recombination. In addition the electron population in the Γ-valley can be enhanced by the heavy n-type doping of Ge^10^. It fills up electronic states in the L-valley up to the Γ-valley. Using such methods, optically pumping^11^ and electrically pumping^12^ Ge lasers have been realized but reliabilities of these lasers still are not satisfactory and therefore another approaches are explored. Alloying Ge with semimetallic α-Sn is one of them^13^,^14^,^15^,^16^,^17^,^18^,^19^,^20^,^21^,^22^. At this moment this approach seems to be the most promising way to achieve the direct gap semiconductor from group IV^23^,^24^.

The pioneering work on GeSn have been done by R. Soref and C. H. Perry^13^ and, later, by He and Atwater^14^. Currently a lot of researchers are focused on the growth of good quality GeSn alloys and devices containing GeSn alloys^15^,^16^,^17^,^18^,^19^,^20^,^21^,^22^. Incorporation of Sn in the Ge crystal reduces the conduction band energy at the Γ-point faster than at the L-point, resulting in a transition from indirect to direct bandgap semiconductor. According to theoretical predictions this transition occurs at Sn concentration of around 5–11% for unstrained GeSn^25^,^26^,^27^,^28^ and depends sensitively on the built-in strain. The first demonstration of optically pumped direct bandgap GeSn lasers has been reported very recently in ref. 29. In this work the authors have obtained lasing from bulk GeSn and determined the material gain for this alloy to be ~100 cm^−1^ at the excitation of ~550 kW/cm^2^.

It is well known that a direct gap type I quantum well is more efficient gain medium than a bulk material and therefore it is very interesting to explore the material gain for GeSn/Ge quantum wells (QWs). So far it has been shown theoretically and experimentally that Ge/Ge_0.92_Sn_0.08_/Ge QW microdisc resonator on Ge/Si (001) is a promising route toward a compact GeSn-based lasers on silicon^30^ but the material gain was not studied in details for GeSn/Ge QW of various contents and widths. Such studies are reported in this paper. To calculate the electronic band structure and material gain for GeSn/Ge QWs the 8-band ***kp*** model has been applied^31^,^32^. It has been found that a positive material gain can be obtained for GeSn QWs grown on Ge. The optimal Sn content of GeSn QW and its width have been determined to be 15% Sn and 12 nm, respectively.

## Results and Discussion

**Figure 1:**
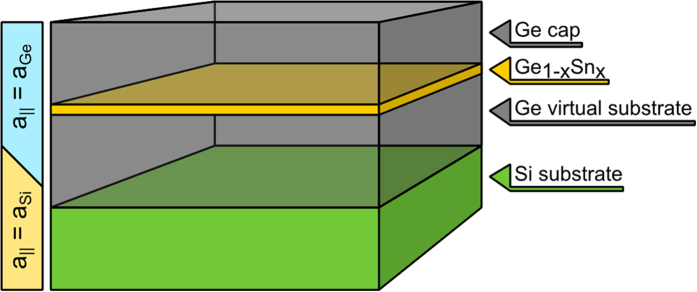
A scheme of a Ge_1−x_Sn_x_/Ge QW deposited on a virtual Ge substrate. The QW is in the region where the in-plane lattice constant 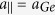.

Figure 1 schematically shows our proposition of a compressively strained Ge_1−x_Sn_x_ QW grown directly on a Ge substrate or a virtual Ge substrate obtained on an Si platform. Technology of such virtual Ge substrates currently is very well developed and they are widely used to deposit good quality Ge_1−x_Sn_x_ layers on it^28^. It is worth noting that this structure differs from the previously considered QWs containing GeSn alloys (i.e., GeSn/SiGeSn QWs^33^,^34^,^35^,^36^,^37^,^38^,^39^) since QW barriers are unstrained and the strain in the GeSn QW is well defined by the lattice constant of Ge. In a GeSn/SiGeSn QW system the strain in the QW region can be balanced by the tuning of the lattice constant of SiGeSn barriers and the relaxed virtual GeSn substrate^34^,^38^. Such approach is very flexible but more challenging from the technological point of view. The structure proposed in this paper is the simplest approach which is very widely realized in III-V lasers. This approach requires two conditions. The first condition is determined by the nature, it is the positive material gain for the GeSn/Ge QW, while the second one is determined by technology, which allows (or not allows) to grow a good quality GeSn material. In this paper the first condition is examined since in case of a promising material gain for GeSn/Ge QWs it can be a strong motivation for growth lasers with the active GeSn/Ge QW region on an Si platform.

A credible model and material parameters are required to determine a material gain for GeSn/Ge QWs. Therefore the two issues are carefully analyzed in this work in order to obtain sound conclusions on the material gain of GeSn/Ge QWs.

In recent years, experimental and theoretical investigations of the electronic band structure for relaxed or unstrained Ge_1−x_Sn_x_ alloys lead to conclusions that the indirect to direct gap crossing is present at ~5–11% Sn^25^,^26^,^27^,^28^. The direct and indirect bandgap in the range of average Sn concentrations can be described by the well-known phenomenological formula


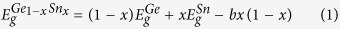


where 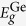 and 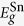 are the bandgaps of Ge and Sn, respectively, and *b* is the bowing parameter. In our calculations this bowing is assumed to be 2.2 and 0.26 eV for direct and indirect bandgap, respectively. It is worth noting that bowing parameters are very crucial for our conclusions of the material gain in GeSn/Ge QWs and therefore the two parameters should be taken very carefully. So far quite different values of bowing parameters were reported in the literature^25^,^26^,^27^,^28^,^40^,^41^,^42^,^43^,^44^,^45^,^46^. From first principles calculations the bandgap bowing parameter for the bandgap at the Γ point was reported to be 1.9 eV^26^, 1.97 eV^28^, 2.06 eV^41^, 2.49 eV^42^, 2.55 eV (2.75 eV for Sn = 12.5%)^25^, and 3.1 eV^27^ while this parameter determined experimentally was reported to be 1.8 eV^28^, 2.42 eV^43^, 1.97–2.61 eV^40^, 2.30–2.84 eV^44^, and 2.8 eV^14^. Experimentally it is more difficult to determine the bowing parameter for the bandgap at the L point of the Brillouin zone. Therefore most of reported bowing parameters for the bandgap at the L point is obtained from theoretical calculations (0.26 eV^28^, 0.89 eV^25^, and 0.9 eV^45^). Recently Tonkikh *et al*.^46^ applied photoluminescence to study GeSn/Ge QWs and obtained that the bowing parameter for bandgap at the L point is 0.17 ± 0.06 eV and 0.8 ± 0.06 eV, respectively, at the assumption of the phonon-related and no phonon-related emission. On the other hand even a negative bowing parameter (b_L_ = −0.11 eV) taken after ref. 40 was used by Sun *et al*.^35^,^36^ to simulate laser diodes containing GeSn/GeSiSn multi QWs. In this context our value of the bowing parameter of 0.26 eV for the indirect gap is in the range of parameters reported in the literature and is fully justified for description of the bandgap at the L point of the Brillouin zone. The same situation is with the bowing parameter for the direct bandgap which has been chosen to our calculations. Since the bowing parameter for the direct bandgap in GeSn alloy is large comparing to other regular semiconductor alloys^47^, a non-linear dependence of the electron effective mass and Luttinger parameters with the increase in Sn concentration is expected for this alloy. Such dependence has been confirmed by Low *et al*.^48^ using the empirical pseudopotential method. However in our calculations we implemented linear interpolation of Luttinger parameters. Such approach is acceptable at the first approximation even if GeSn is described with the large bandgap bowing. Moreover such approach is more safe in this case taking into account the narrow bandgap of GeSn and quite large range of bowing parameters reported in the literature for this alloy. Additionally, it has been shown that the Bir-Pikus theory^49^ can be applied to description of the strain-related shifts in the conduction and valence bands in this alloy^28^,^50^. This theory has been used by us to calculate the quantum confinement potential for electrons and holes in strained GeSn/Ge QWs.

**Figure 2:**
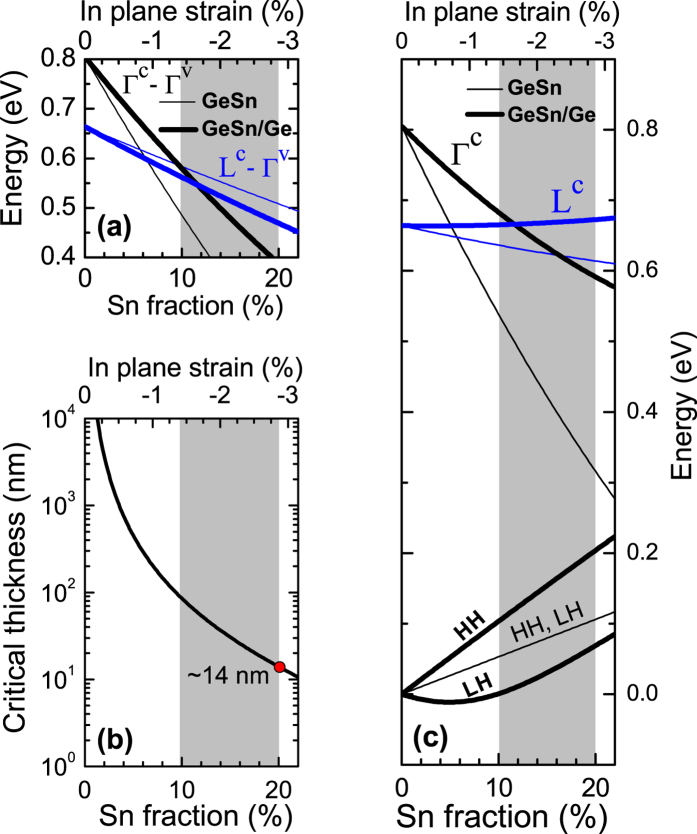
The direct (black lines) and indirect bandgap (blue lines) in unstrained Ge_1−x_Sn_x_ (thin lines) and coherently strained Ge_1−x_Sn_x_ on Ge (thick lines). (**b**) Critical thickness of Ge_1−x_Sn_x_ layer deposited on Ge substrate. (**c**) The HH/LH band and conduction band at the Γ and L point in unstrained Ge_1−x_Sn_x_ (thin lines) and coherently strained Ge_1−x_Sn_x_ on Ge (thick lines).

Figure 2(a) shows the content dependence of direct and indirect gaps for unstrained (thin lines) and strained (thick lines) Ge_1−x_Sn_x_. In addition, the critical thickness of Ge_1−x_Sn_x_ layer, which is coherently strained on Ge, is calculated according to refs 51,52 and shown in Fig. 2(b). From this figure it can be concluded that Ge_1−x_Sn_x_ alloy with x >10% is interesting to be considered as a gain medium due to its direct gap. On the other hand Sn concentration cannot be too high because of the compressive strain in this layer which should be rather smaller than 3%. The range of GeSn content resulting from these two conditions is shown by a grey background in Fig. 2. It is worth noting that Sn concentration for which a direct gap is expected in compressively strained GeSn strongly depends on the bandgap bowing at the Γ and L point of the Brillouin zone as well as pressure coefficients for the conduction band at these points. In some ranges of these parameters even no direct gap can be expected for GeSn strained on Ge^45^. It means again that material parameters have to be chosen very carefully in order to perform reliable calculations for this QW system.

**Figure 3:**
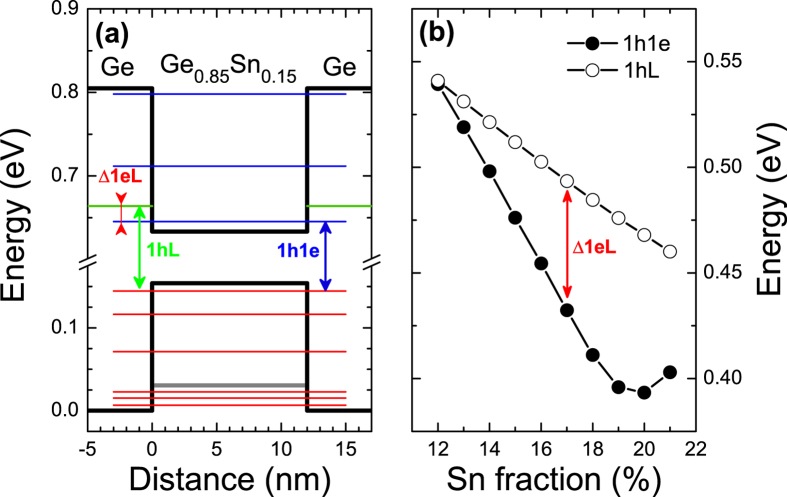
(**a**) Electron, heavy hole (black lines) and light hole (grey line) potentials for the 12 nm wide Ge_0.85_Sn_0.15_/Ge QW together with the electron (blue lines) and hole (red lines) confined states energies and the L valley minimum energy in Ge barriers (green line). (**b**) The energy of the 1 h1e transition in QW (black circles) and the energy difference 1 hL between the first electron level in QW and L valley in Ge barriers (open circles) obtained for Ge_1−x_Sn_x_/Ge QWs of various Sn concentrations and the width corresponding to the critical thickness at a given Sn concentration.

Regarding the application of GeSn/Ge QWs as the gain medium in lasers, the type I bandgap alignment for electrons and heavy holes is required. Taking into account the valence band offset between Ge and Sn determined in ref. 53 and adopting the model-solid theory^54^ to calculate the valence band position in Ge_1−x_Sn_x_ alloy, the quantum confinement potential for electrons and holes has been determined. Figure 3 shows potential profiles for the Ge_0.85_Sn_0.15_/Ge QW together with energy levels calculated for this QW. It is visible that such QW is type I with a strong quantum confinement in both the conduction and the valence band. However the L valley in Ge barriers are located very close to the conduction band minimum in QW region. It means that energy difference between the L valley in the Ge barriers and the first electron level in QW (Δ1eL) is a parameter which characterizes the utility of a GeSn/Ge QW in laser applications (this energy is marked by the red arrow in Fig. 2). Figure 3(b) shows an energy of the ground state transition in Ge_1−x_Sn_x_/Ge QWs of various contents and the width corresponding to the critical thickness for given Ge_1−x_Sn_x_ content (solid black points) together with the energy between the L valley in Ge barriers and the first heavy hole subband in the Ge_1−x_Sn_x_/Ge QWs (open points). In this figure the energy difference between open and solid black points corresponds to the Δ1eL energy. It has been found that in the range of Sn > 10% the L valley in Ge barriers are located above the first electron subband in Ge_1−x_Sn_x_/Ge QW and the Δ1eL energy increases with the increase in Sn concentration, see Fig. 3(b). Such conditions are very favorable for achieving an inversion of carrier population in the QW region and therefore it is very interesting to calculate the material gain for Ge_1−x_Sn_x_/Ge QWs with the contents of 0.1 < × < 0.2 and various widths.

**Figure 4:**
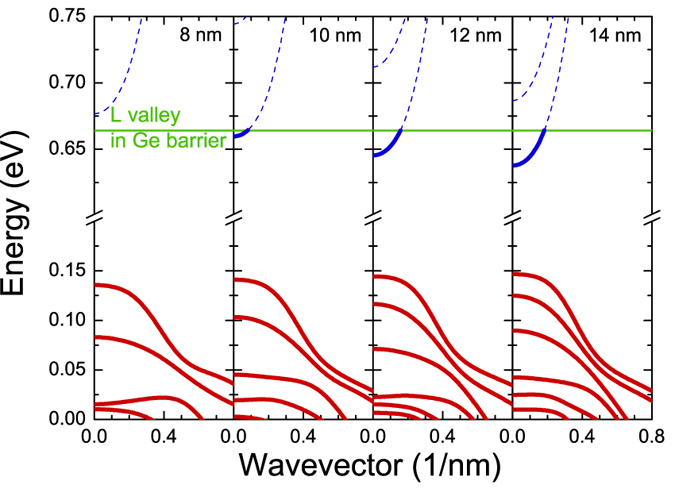
The electronic band structures along the [100] direction for 8, 10, 12 and 14 nm wide Ge_0.8s_Sn_0.1s_/Ge QWs.

An 8-band ***kp***-model is widely used to calculate the electronic band structure near the center of Brillouin zone for direct gap III-V semiconductor crystals and QWs^31^,^32^. The same approach has been applied to calculate the electronic band structure near the center of Brillouin zone for Ge_1−x_Sn_x_/Ge QWs. Such calculations for Ge_0.85_Sn_0.15_/Ge QWs of various widths are shown in Fig. 4. It is clearly visible that the quantum confinement for heavy-holes is strong and a significant mixing of heavy-hole and light-hole subbands is observed only for higher subbands at large ***k*** vectors (***k*** > 0.2 nm^−1^). It means that the valence band structure is very promising from the viewpoint of transverse electric (TE) mode of the material gain. In order to discuss the dispersion of electron subbands, the position of the minimum of the L valley in Ge barriers are plotted in this figure. This position is very important for proper calculations of the material gain since electrons can occupy the L valley as well. For the QWs shown in Fig. 4 the minimum of conduction band in QW region is located below the L valley in Ge barrier when the QW width is larger than 8 nm. As earlier mentioned such conditions are very promising from the viewpoint of inversion of carrier population in the QW region. For an opposite situation (i.e., the conduction band minimum in the QW region above the L valley in Ge barrier) the inversion of carrier population can be not present or can be expected at very high carrier concentrations. In our calculations of the material gain for Ge_1−x_Sn_x_/Ge QWs the effect of the L valley is taken into account.

**Figure 5:**
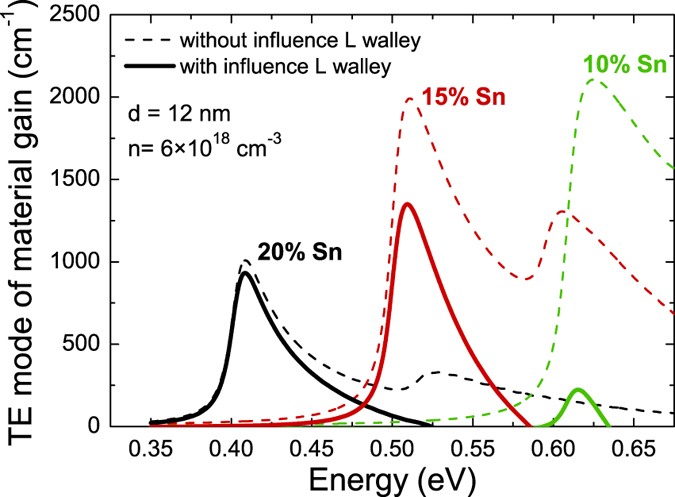
Material TE gain for 12 nm wide QWs with 20%, 15%, 10% Sn contents. There is TE gain calculated with the influence of the L valley (solid lines) and without it (dashed lines).

Figure 5 shows TE mode of the material gain for 12 nm wide Ge_1−x_Sn_x_/Ge QWs of various contents calculated with (solid lines) and without (dashed lines) consideration of occupation of the L valley. The transverse magnetic mode of the material gain is not consider for this QW since its spectral position is at higher energy due to compressive strain in this QW and thereby is not important for lasing. In Fig. 5 it is clearly visible that differences between solid and dashed lines are very large for QW with 10% Sn concentration and significantly smaller but still present for QW with 20% Sn. It means that the occupation of the L valley by carriers plays a significant role for QWs with lower Sn concentrations i.e., a situation where the L valley in Ge barriers are located closer to the CB minimum in QW region. It is worth noting that such a problem does not exist in calculations of the material gain for most of direct gap III-V QWs^33^. In order to perform the material gain engineering in GeSn/Ge QWs we have to take influence of the L valley into account since without this effect conclusions on the optimal content and width of GeSn/Ge QWs for laser applications can be invalid. Therefore our calculations, which are discussed in the next part of this paper, includes the occupation of L valley by carriers.

**Figure 6:**
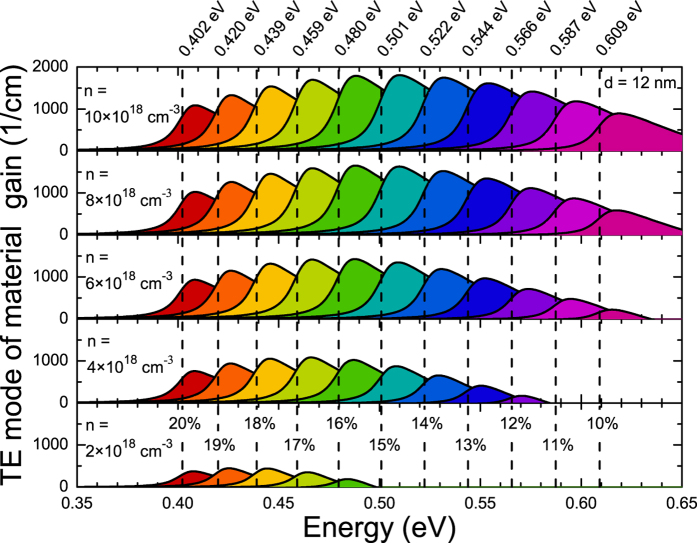
TE mode of the material gain for 12 nm wide Ge_1−x_Sn_x_/Ge QWs with various Sn concentrations and carrier densities *n*. Vertical dashed lines show the energy of the fundamental QW transition for each QW.

**Figure 7:**
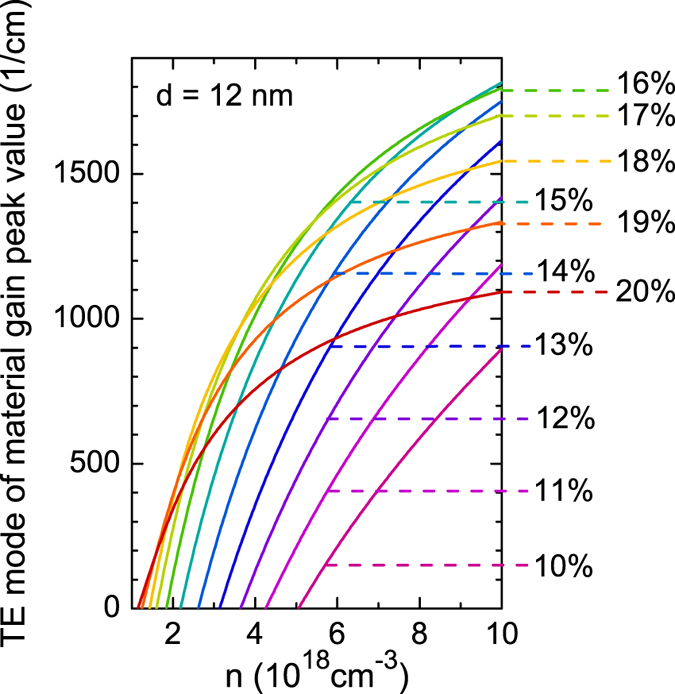
TE mode of the material gain at the peak position obtained for various Sn concentrations for 12 nm wide Ge_1−x_Sn_x_/Ge QWs with various Sn concentrations.

Figure 6 shows the transverse electric (TE) mode of the material gain for 12 nm wide Ge_1−x_Sn_x_/Ge QWs of various Sn contents calculated for different carrier concentrations. The energy of the ground state transition in each QW is marked by a vertical dashed line together with the Sn concentration corresponding to this QW. First, it is visible that each peak of the material gain is observed a few meV above the corresponding to it ground state transition and does not shift significantly with the increase in carrier concentration. Moreover it is visible that large material gain (~500 cm^−1^ at the peak position) is observed even for low carrier concentration (n = 2 × 10^18^ cm^−3^) for Ge_1−x_Sn_x_/Ge QWs with larger Sn concentration (Sn >15%). For the QWs with Sn < 15% the positive material gain is also observed for carrier concentration n >2 × 10^18^ cm^−3^. Gain dependency on carrier concentration is better illustrated in Fig. 7 where the material gain at the peak position is plotted for QWs studied in Fig. 6. From this figure it is easy to conclude that taking the maximal material gain at the minimal carrier concentration into account the most optimal are Ge_1−x_Sn_x_/Ge QWs with 15–18% Sn.

**Figure 8:**
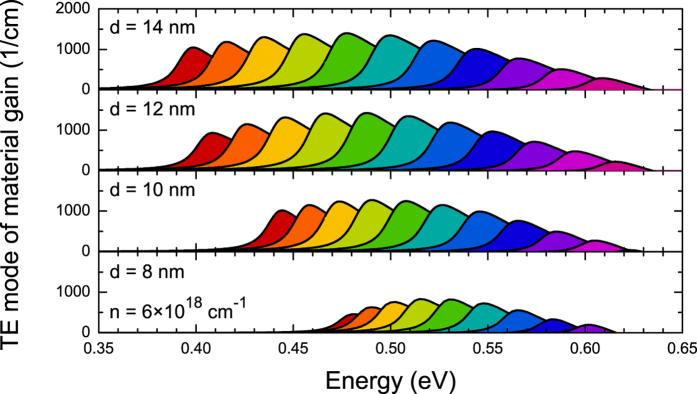
TE mode of the material gain for Ge_1−x_Sn_x_/Ge QWs with various widths and Sn concentrations.

**Figure 9:**
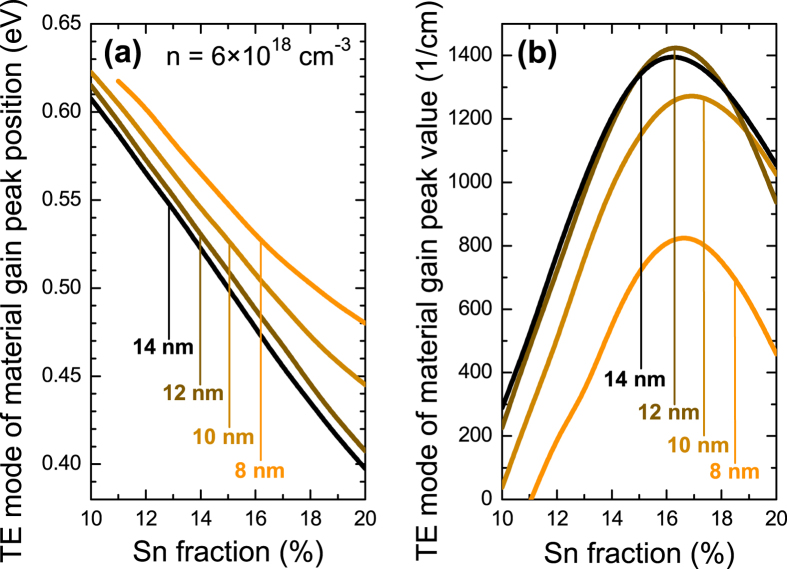
The peak energy (**a**) and value (**b**) obtained for Ge_1−x_Sn_x_/Ge QWs with various widths and Sn concentrations.

The influence of QW width on the material gain is analyzed in Figs 8 and 9. In this case the material gain is calculated for carrier concentration of 6 × 10^18^ cm^−3^. The maximal gain is observed for Ge_1−x_Sn_x_/Ge QWs with ~15% Sn, see Fig. 9(b). With the increase in QW width from 8 nm to 12 nm the material gain increases while the further increase in QW width leads to a decrease of the material gain, see also Fig. 9(b). The spectral position of the material gain in 14 nm wide Ge_1−x_Sn_x_/Ge QWs can be tuned from ~1.9 to ~3.1 μm by an increase in Sn concentration from 10 to 20%. In addition it is visible that the gain peak position can be tuned by changing the QW width. It means that such QWs have a potential applications in gas sensing where currently GaSb-based lasers are applied^32^,^55^. So far a positive gain has been predicted to be present for GeSn QWs with GeSiSn barriers^33^,^34^,^35^,^36^,^37^. In this case GeSiSn barriers enhance the quantum confinement and can be used to compensate the compressive strain in GeSn quantum region^34^. However GeSn/GeSiSn QW system is very challenging from the viewpoint of the growth as well controlling the alloy content and the structural/optical quality for such QWs. GeSn/Ge QW system, which is considered in this article, can be simpler since such system requires only good quality GeSn material with relatively large Sn concentration (10–20%). Therefore we believe that GeSn/Ge QWs with large Sn concentrations are very interesting to explore experimentally since they are a promising gain medium for mid infrared lasers integrated with the Si platform.

An addition material gain engineering in Ge_1−x_Sn_x_/Ge QWs can be achieved via n-type doping the QW region. As mentioned in the introduction so far such approach has been applied to Ge lasers^10^,^11^,^12^. In case of the GeSn/Ge QW it is expected that the range of Sn concentration, for which a positive material gain is expected, can be a little bit broadened by n-type doping of the QW region and/or Ge barriers near the QW region. Such doping is able to fill L valley in Ge barriers and thereby enhance the material gain. On the other hand it is well known that the high carrier concentration (>10^18^ cm^−3^) induces a free carrier absorption in the mid-infrared spectral region. This effect is neglected in our calculations since we consider undoped Ge barriers and the expected emission wavelengths (~2–3 μm) are not very long, however for GeSn QWs with doped Ge barriers the effect of free carrier absorption can be important.

## Conclusions

It is shown that compressively strained Ge_1−x_Sn_x_/Ge QWs of various contents (0.1 ≤ × ≤ 0.2) and widths (8 nm ≤ d ≤ 14 nm) grown on Ge substrates (or templates) have a very promising material gain for laser applications. Taking into account the maximal material gain at the minimal carrier concentration the most optimal are ~12 nm width Ge_1−x_Sn_x_/Ge QWs with ~15–18% Sn concentration. Reduction of Sn concentration below 15% decreases the material gain due to unfavorable population of carriers in the conduction band (i.e., carriers occupy L valley in Ge barrier instead of QW region at the Γ point), while the increase of Sn concentration above 20% causes a decrease of the material gain due to the required reduction of QW width below the critical thickness of GeSn deposited on Ge. The energy difference between L valley in Ge barrier and the first electron subband in the QW region (i.e., the Δ1eL energy) very strongly decreases with the reduction of QW width. For narrow QWs (width <8–10 nm at Sn>20%) carriers occupy L valley in Ge barrier instead of QW region at the Γ point and hence this range of Sn concentration is unfavorable.

## Methods

### 8 band *kp* Hamiltonian

For unstrained GeSn the 8 band ***kp*** Hamiltonian is given below


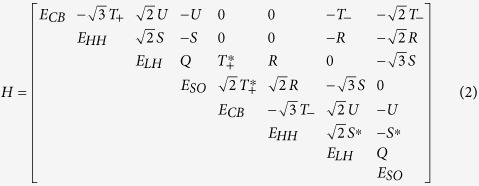


The matrix elements in this Hamiltonian are the following:


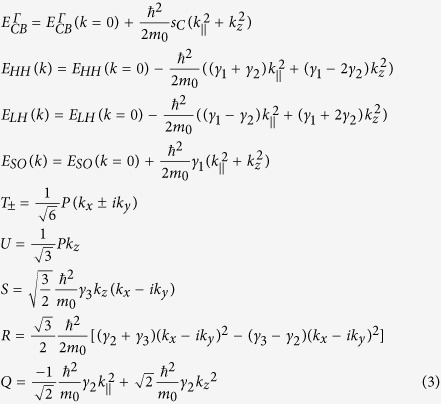


where the subscripts CB, HH, LH, and SO stand for the conduction (Γ point), heavy-hole, light-hole, and spin-orbit split off bands, respectively. 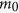 is the electron mass, 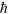 is the Planck constant divided by 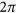, and 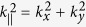. *P* is the Kane matrix element defined as 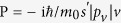, where 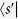 indicates a CB Bloch state of s-like symmetry and 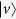 is a VB p state with character *P* = −*i*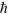/*m*_0_ 〈*s*′*p*_v_*v*〉. Since the CB is treated exactly in this Hamiltonian, the Luttinger parameters are modified, i.e. the parameters 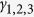 in the matrix elements are replaced by 
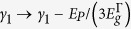
 and 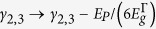, where 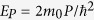 is the Kane matrix element expressed in energy units. The term 
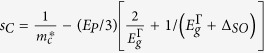
 replaces 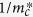 and is associated with the CB nonparabolicity. The energy scale is assumed such that at the Γ point for unstrained Ge 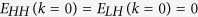, 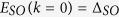, and 
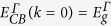
, where 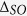 and 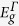 are, respectively, the spin-orbit splitting and the energy gap for Ge.

### Material parameters and strains

The band offset between Ge and GeSn is calculated for unstrained materials according to the model-solid theory^54^ with the band offset between Ge and Sn equal 0.53 eV^53^. In this case the standard and most effective approach to band offset calculations has been applied^25^,^54^. In this approach to obtain the energy scale relative to the vacuum level slabs consisting of 32 atomic layers in (100) direction, for 8 different compositions, based on the relaxed special quasirandom structures have been constructed. The thickness of the slab was chosen after a careful convergence study. The total Kohn-Sham potentials of said slabs were then confronted with their bulk counterparts. The potential of the bulk structure was shifted to match the potential of the center of the slab, where the influence of the surface was negligible and then both potentials were shifted for the vacuum potential to be set at 0 eV. The total shift was then added to the bulk band structure to obtain the correct energies of the bands relative to vacuum^53^. The strain-related shifts for CB and VB are calculated using the Bir-Pikus Hamiltonian^49^. These strains modify the quantum confinement potential for electrons and holes as shown in our previous papers^31^,^32^. In the QW region the matrix elements of the 8-band ***kp*** Hamiltonian are modified as


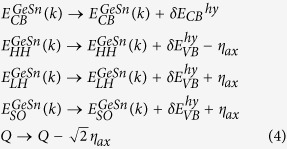


where 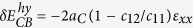, 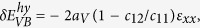 and 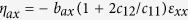 describe influence of the hydrostatic and shear strain components on the band structure. *a*_*c*_ and *a*_*v*_ are the hydrostatic deformation potentials for CB and VB, respectively; *c*_11_ and *c*_12_ are elastic constants, *b*_*ax*_ is the axial deformation potential and ε_*xx*_ is the in-plane strain in the GeSn layer. For GeSn QWs grown on a Ge substrate this strain is defined by the lattice parameters of GeSn and Ge: 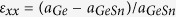 Material parameters for Ge_1−x_Sn_x_ are determined using linear interpolation between parameters of relevant binary compounds. Material parameters of Ge and Sn used in our calculations are taken after refs 28,34 and they are given in Table 1.

### Material gain

The optical gain for GeSn/Ge QWs is calculated for a given carrier density, which determines the quasi-Fermi levels 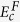 and 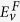 for the conduction and valence bands, respectively, and vice versa. The carrier density in a band in the QW is given by integration of product of the density of states, 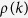, and the occupation probability of carriers (i.e. the Fermi-Dirac distribution) over the entire band. The Fermi-Dirac distribution for electrons (*f*_*c*_) and holes (*f*_*v*_) in the QW is given by Eqs (5) and (6)


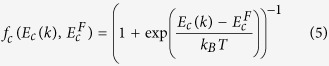



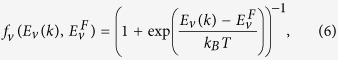


where *k*_*B*_ is the Boltzmann’s constant and *T* is temperature. The carrier density in conduction (*N*) and valence (*P*) band is obtained by


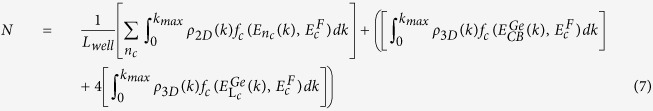



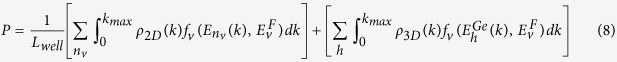


First terms on the right hand sides of equations (7) and (8) are related to the 2-D Fermi gases described by electronic band structure of the QW obtained in the planewave expansion of the 8 ***kp*** model, while following ones are related to 3-D Fermi gases, which are described by electronic band structure of unstrained bulk Ge crystal calculated using the 8***kp*** model. *L*_*well*_ is the QW width. The third term in integral (7) takes account of 3-D electron Fermi gas in L valley, where 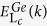 is parabolic approximation of this band in bulk crystal. It is shifted to the center of Brillouin zone in order to simplify the quasi-Fermi level calculations and hence the factor 4 appears at this term (this simplification is equivalent to eight L valleys at the border of the Brillouin zone). Transitions related to this valley are omitted in the gain calculations. Further for a non-parabolic bands, integration is carried out in a ***k*** space with the density of states determined from the ***kp*** calculations. *k*_*max*_ is the integration limit determined by convergence of the integrals (7) and (8).

A conventional method based on the relaxation time approximation convoluted with a Lorentzian function (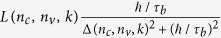. where 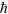 is Plank’s constant and Δ is the proper energy difference) with a proper broadening time (*τ*_*b*_ = 0.1 ps) was used to calculate the gain spectra^31^,^32^. In this approximation the transverse electric (TE) and transverse magnetic (TM) gain is given by Eq. (9)


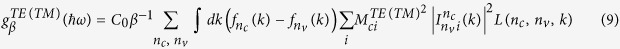


where 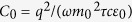 (*q* - elementary charge; *m*_0_- electron mass; *ω*– angular frequency, *τ*- time constant; *c*– speed of light; *ε*_0_- dielectric constant), *i* represents the heavy and light hole subbands, *β* in this case is the propagation constant of the TE and TM mode, 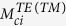 is the matrix element of TE (TM) mode, and 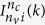 is the overlap integral. In our calculations we do not consider many-body effects as well Auger processes. It means that our approximation works well for carrier concentration lower than ~8 × 10^18^ cm^−3^. Despite this fact in the framework of this approach we are able to identify the range of QW content and width, which is the most optimal from the viewpoint of positive material gain and its spectral position.

## Additional Information

**How to cite this article**: Mączko, H. S. *et al*. Material gain engineering in GeSn/Ge quantum wells integrated with an Si platform. *Sci. Rep.* **6**, 34082; doi: 10.1038/srep34082 (2016).

## Figures and Tables

**Table 1:** Material parameters for ge and sn.

Parameters	Ge	α-Sn
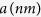	0.56573^a^	0.64892^a^
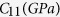	128.53^a^	69.00^a^
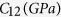	48.26^a^	29.30^a^
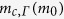	0.038^a^	0.058^a^
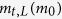	0.0807^a^	0.075^a^
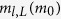	1.57^a^	1.478^a^
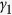	13.38^a^	−14.97^a^
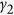	4.24^a^	−10.61^a^
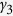	5.69^a^	−8.52^a^
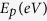	26.3^a^	24.0^a^
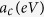	−8.24^a^	−6.00^a^
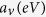	1.24^a^	1.58^a^
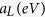	−1.54^a^	−2.14^a^
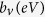	−2.9^a^	−2.7^a^
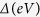	0.30^b^	0.67^b^
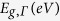	0.805^b^	−0.41^b^
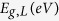	0.664^a^	0.092^a^
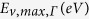	0.00^c^	0.53^c^
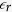	16.2^a^	24.0^a^

^a^Ref.^34^.

^b^Ref. 28

^c^Ref. 53.
